# Sonoelastography evaluation in the diagnosis of endometrial pathology combined with chronic endometritis in infertile women

**DOI:** 10.25122/jml-2021-0358

**Published:** 2022-03

**Authors:** Iryna Kostiantynivna Orishchak, Oksana Mykhailivna Makarchuk, Nataliia Ivanivna Henyk, Oksana Mykolaivna Ostrovska, Halyna Myroslavivna Havryliuk

**Affiliations:** 1. Department of Obstetrics and Gynaecology of Postgraduate Education, Ivano-Frankivsk National Medical University, Ivano-Frankivsk, Ukraine; 2. Department of Obstetrics and Gynaecology named after I. Lanovyi, Ivano-Frankivsk National Medical University, Ivano-Frankivsk, Ukraine

**Keywords:** endometrial hyperplasia, benign ovarian cysts, chronic endometritis, sonoelastography

## Abstract

Endometrial pathology, including hyperplastic processes in the structure of reproductive disorders, occupies one of the leading places along with inflammatory diseases of the pelvic organs, contributing to infertility in 80% of cases and irregular menstrual cycle in 40–43%. This study aims to optimize the diagnostic algorithm in patients with endometrial hyperplasia combined with chronic endometritis and determine qualitative indicators of compression sonoelastography in patients with endometrial pathology and infertility. A comprehensive clinical and laboratory examination of 90 infertile patients aged 25 to 45 years with endometrial hyperplasia combined with chronic inflammation, retention cysts, and benign ovarian tumors was carried out. The results of clinical-laboratory and complex ultrasound examination with compression sonoelastography were compared with the data of pathomorphological and immunohistochemical studies. A high percentage of pelvic inflammatory disease (55.0%), benign lesions of the cervix (67.5%), hyperplastic processes of the myometrium (37.5%), an increasing number of polyps by 2.9 times, leiomyomas and adenomyosis – by 2.3 times (p<0.05) was established. In the case of a combination of endometrial hyperplasia and ovarian cysts, a high percentage of comorbidity of gynecological pathology is verified (37.8%), and the use of compression sonoelastography allows to establish class II and class III elastograms in 91.1% of cases which characterize benign endometrial lesions, reduce the number of false-positive results in 95.6% of cases, correctly interpret the nature of pathological changes and increase the sensitivity of ultrasound techniques.

## INTRODUCTION

Recent publications demonstrate the increasing frequency of hyperplastic processes (HP) of the endometrium, accompanied by an increase in the proportion of surgical interventions, emphasizing the social aspect of the problem [1–3]. At least 52–74% of women who received hormone therapy experience a recurrence and persistence of the disease [2, 4–7]. The literature emphasizes the comorbidity of HP of reproductive organs and their combination with uterine leiomyoma, adenomyosis, benign ovarian tumors, pathology of the mammary glands, which is often preceded by pelvic inflammatory disease. Comorbidity of gynecological nosology contributes to the resistance of conservative treatment, high recurrence rate, and initiation of malignancy mechanisms, especially in the long course of the disease [2, 4–7]. Low treatment efficiency and prevention measures are due to the lack of etiological orientation as a result of insufficient understanding of the leading mechanisms of proliferative processes.

Endometrial pathology, including HP in the structure of reproductive disorders, occupy one of the leading places, along with pelvic inflammatory disease [4–7], contributing to infertility in 80% of cases and menstrual disorders in 40–43% [1, 3]. Both the state of the endometrium and chronic forms of pelvic inflammatory disease, in general, require a personalized approach and management optimization at the stage of periconceptional care, along with miscarriage and diminished ovarian reserve, changes in structural parameters of endometrium and ovaries, and chronic inflammatory process that affect the risks of subfertility and failure of assisted reproductive technologies (ART) [6, 8–10]. Hyperplastic processes that negatively affect fertility include endometrial polyps diagnosed in 16–25% of studies, where 46% are atrophic polyps, 19% are hyperplastic, adenomatous, and 2% to 0.6% are mixed ones, respectively [6, 9, 10].

Recent molecular-biological discoveries expand the idea of the pathogenesis of hyperplastic processes of the endometrium and go beyond the traditional scientific concept of relative and absolute hyperestrogenism [2, 6, 7, 9]. Today, HP of the endometrium is considered a polyetiological pathological process, the development and progression of which is facilitated by many factors, including the patient's age and the premenopausal period when hormonal changes create the conditions for the development of hyperplastic processes [1–3]. Among the leading factors, the following disorders should be noted: extragenital diseases (thyroid dysfunction, overweight, pathology of the gastrointestinal tract, cardiovascular disease) and comorbidity of gynecological pathology (leiomyoma, adenomyosis, polycystic ovary syndrome, benign ovarian tumors [9, 11–14]. It is necessary to emphasize recent data on the growth of chronic endometritis (CE) as one of the factors in the development of HPE [11, 12, 15, 16]. Against the background of pelvic inflammatory disease and hormonal imbalance, there is a probability of developing pathological processes in the ovaries [1–3, 13]. Studies show the development of tumor-like retention cysts and true ovarian tumors in this category of patients [8, 17, 18], and their share increases significantly after 40 years from 15% to 42%; the retention cysts and tumor-like lesions occur in 4–19% of observations [13, 18–20].

Modern scientific advances allow the use of highly informative methods for diagnosing uterine infertility factors, including ultrasound (US), hysteroscopy and papillae biopsy, immunohistochemical and immunopathological techniques, bacteriological and DNA PCR, but morphological identification remains the gold standard [21–23]. In recent years, the so-called micromanipulation technologies, such as endoscopy have been actively introduced, significantly increasing the accuracy of diagnosis and effectiveness of ART programs. The search for an optimal combination of different ultrasound techniques (colour Doppler mapping, energy Doppler mapping, and 3D mode) for maximum accurate visualization of the structural parameters of the endometrium is currently in progress [24–26]. In recent years, according to the results of Belozerova (2016), compression sonoelastography has been used to improve the informativeness of the ultrasound picture of endometrial pathology, which allows supplementing the standard approach, especially in the case of combined HPE pathology with adenomyosis and uterine leiomyoma, which often reduces the diagnosis by 7–10% [14, 24, 25, 27, 28].

The expediency of detailing the pathogenetic aspects of HPE is due to the improvement of diagnosis and optimization of therapeutic programs related to the correction of various components of the immune system and assessment of the role and impact of infectious factors [29, 30]. Analysis of scientific reviews suggests that a comprehensive approach to the proliferative lesions of the endometrium will allow the development of an effective algorithm for monitoring and supporting this category of women. Despite the significant attention of researchers, the full potential of compression sonoelastography in the diagnosis of various pathological conditions of the endometrium has not been fully studied; no algorithm has been developed for comprehensive ultrasound examination using compression sonoelastography for treatment of patients with HPE, which determined the relevance of this scientific work.

This study aimed to optimize the diagnostic algorithm in patients with endometrial hyperplasia combined with chronic endometritis and determine qualitative indicators of compression sonoelastography in patients with endometrial pathology and infertility.

## MATERIAL AND METHODS

The study was conducted on the clinical basis of the Department of Obstetrics and Gynaecology of Postgraduate Education of Ivano-Frankivsk National Medical University, Gynaecology Department of the Regional Perinatal Center in 2019–2021. A comprehensive clinical and laboratory examination of 90 infertile women aged 25 to 45 years with endometrial hyperplasia in combination with chronic inflammation was carried out.

Inclusion criteria were: age from 25 to 45 years, infertility, morphologically confirmed endometrial hyperplasia without atypia, an immunohistochemical marker of inflammation CD 138+, and informed voluntary consent of patients to undergo the necessary diagnostic measures. Exclusion criteria were: atypical endometrial hyperplasia and malignancy, large uterine fibroids, external genital and extragenital endometriosis, grade II-III adenomyosis, acute pelvic inflammatory disease, severe extragenital diseases, the patient's refusal to participate in the study.

In order to clarify the condition of the endometrium, the hysteroscopy was performed for diagnostic and therapeutic purposes, followed by histological and immunohistochemical examination of the mucosal biomaterial. Based on the clinical course, data of genital status, immunohistochemical and pathomorphological conclusions, the following study groups were formed: group 1 consisted of 40 patients with HPE on the background of chronic endometritis and retention cysts and benign ovarian tumors; group 2 – 50 patients with HPE on the background of chronic endometritis. The control group included 30 women from the contingent of relatively healthy patients without gynecological pathology.

Assessment of anamnestic data was performed on a specially designed questionnaire which consisted of 126 items. Clinical research methods included a general examination of organs and systems, analysis of gynecological status, instrumental and laboratory research methods, transvaginal ultrasound scanning, and qualitative parameters of sonoelastography. Transvaginal ultrasound scanning was performed using a digital diagnostic ultrasound scanning system HITACHI ALOKA with a built-in elastography program using an endocavitary sensor with a frequency of 8-4 MHz: uterine size, structural features of the endometrium, size and structure of the median M-echo, ovarian structural parameters and qualitative indicators of sonoelastography were evaluated. To determine the parameters of blood flow in the vessels of the uterus and endometrium and pathological formations, the technique of colour Doppler and energy mapping was used. To evaluate the obtained elastograms, the scale of elastographic images adapted for gynecology was used; the results of a comprehensive ultrasound examination with compression sonoelastography were compared with the data of pathomorphological studies. The investigation and measurements were performed in a gynecological mode; the measurements were performed with a sensor in the area located close to the uterine body wall with an assessment of tissue stiffness in three standard areas of interest according to the shape and size (Q-Box) along the muscle fibers of the myometrium. The indicators were determined in real-time. All the received information was stored in device memory for further processing. The mean value (Emean), maximum value (Emax), and standard deviation (SD) were used for analysis. Then the average value for each indicator was calculated (for Emean, Emax and SD). Based on the obtained results, a colour map was formed in the window of interest, which was superimposed on the image in B-mode. The toughest tissue was mapped in blue, and less tough tissue was mapped in red. Medium-tough tissues were mapped in green and yellow tones. Reproduction of compression sonoelastography was evaluated by two independent specialists in ultrasound diagnostics according to the Kappa statistic measure; clinical significance was evaluated according to a 3-point scale: 1 point – sonoelastographic image allows to clarify the diagnosis; 2 points – sonographic image allows to obtain additional information; 3 points – sonographic image allows to get data for a correct diagnosis. Statistical processing of the obtained data was performed using Stat Soft Statistica v6.0 and Microsoft Excel 97 application packages.

## RESULTS

We examined 90 patients aged 33.20±6.60 years with infertility. The body mass index of women ranged from 24.6±4.8 kg/m^2^ to 33.7±2.6 kg/m^2^. Primary infertility was diagnosed in every fourth patient (23–25.6%).

Regarding benign tumors and ovarian retention cysts in Group 1, the largest share was follicular cysts (15–37.5%), corpus luteum cysts (7–15%), endometriomas (5–12.5%), and epithelial cysts (13–32.5%).

**Figure 1.**
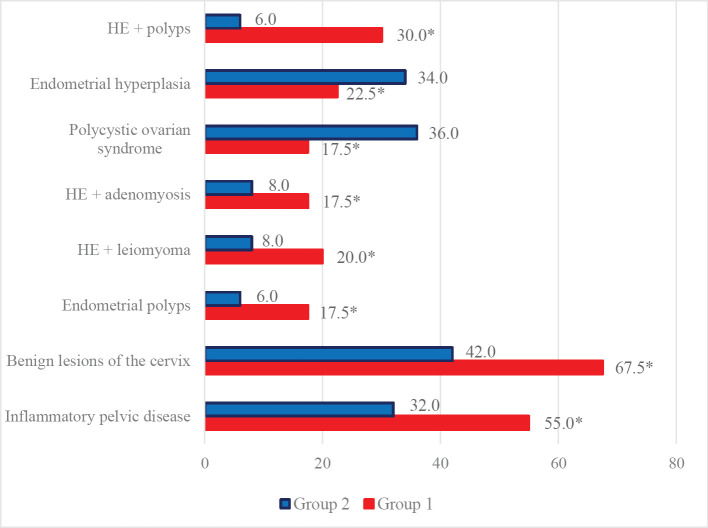
Structure of gynecological pathology in the studied groups, n=90%. Note* – the difference is significant for the data from Group 2, p<0.05.

In the structure of concomitant gynecological diseases in Group 1, pelvic inflammatory disease predominates in half of the observations (22–55.0%) against a third (16–32.0%) in Group 2 (p<0.05), benign lesions of the cervix (27–67.5% *vs.* 21–42.0%, respectively, p<0.05), hyperplastic processes of the myometrium (15–37.5% *vs.* 8–16.0%, respectively, p<0.05) and hyperplastic processes of the mammary glands which are evidence of the systemic nature of combined gynecological pathology (Figure 1).

Combinations of hyperplastic processes of the myometrium (adenomyosis, uterine leiomyoma) were observed in more than a third of patients in Group 1. There is a clear relationship of HP with such factors as hereditary predisposition, puberty disorders, high frequency of intrauterine interventions, pelvic inflammatory disease, and low somatic health indicators. In the analysis of gynecological pathology, the frequency of endometrial polyps in Group 1 was 2.9 times higher than in patients from Group 2 (p<0.05), which should be associated with the persistence of risk factors and resistance to classical therapeutic approaches. Besides, according to a comprehensive ultrasound study, in 15 patients from both study groups (16.7%), a combination of HE and endometrial polyps was revealed, polyposis was diagnosed in 10 patients (11.1%), a combination of HE with uterine leiomyoma was noted in 12 cases (13.3%), 11 cases (12.2%) had adenomyosis. The diagnosis was somewhat difficult in 6 women (6.7%) due to the concomitant gynecological pathology and questionable ultrasound data.

Despite the lack of significant deviations in the proportion of leiomyoma and adenomyosis compared to the control group (4–13.3, p>0.05), there was an increase of 2.3 times in nosology frequency in Group 1 compared to Group 2. The total frequency of hyperplastic processes of the myometrium in Group 1 was 15 (37.5%) compared to 8 (16.0%) (p<0.05). Moreover, leiomyoma and adenomyosis were considered independent factors in the combination of HE and polyposis (risk ratio 3.15%, confidence interval 1.17–8.48, p<0.05). Hyperplastic endometrial processes in their combined variant (endometrial hyperplasia, uterine leiomyoma, adenomyosis, and benign ovarian tumors) were revealed in 34 cases (37.8%). Notably, the structural parameters of the ovarian tissue, characteristic of polycystic ovarian syndrome, were verified mainly in Group 2, which is associated with the pathogenetic aspects of endometrial hyperplasia.

Endometrial hyperplasia without atypia and combined pathology were found in 9 patients (22.5%) from Group 1, while in Group 2, it was revealed in 17 cases (34.0%), *i.e.*, 1.5 times more often (p<0.05). Combined endometrial pathology, namely endometrial hyperplasia without atypia, is combined with chronic endometritis. It was confirmed in all samples of both study groups where the diagnosis was confirmed by positive verification of the immunohistochemical marker of inflammation CD-138 +, as well as by lymphoid infiltration in 82 cases (91.1%), vascular sclerosis – in 24 (26.7%), stromal sclerosis – in 46 (51.1%) and by lymphoid follicles – in 26 cases (28.9%), according to the results of the pathohistological examination. In patients from Group 1, the number of CD-138 + plasma cells was significantly increased; histological examination showed diffuse plasmacytic infiltration of the endometrial stroma, severe periglandular sclerosis of the stroma, and monomorphic type of glands, which is an immune-histomorphological confirmation of chronic endometritis with reactive endometrial hyperplasia. According to the immunohistochemical study, patients from Group 2 had focal accumulations of plasma tissues CD138+ in small quantities, indicating the development of moderate chronic inflammation, which eventually acquires the features of chronic endometritis. Considering the histological examination, such inflammatory changes should be regarded as secondary (reactive) endometritis in the case of estrogen-dependent endometrial hyperplasia. The results of the morphological examination are presented in Table 1.

**Table 1.** Morphological data from endometrial biopsy.

Morphological changes of the endometrium	Group 1, n=40		Group 2, n=50		Control group, n=30	
	abs (numb)	%	abs (numb)	%	abs (numb)	%
**Stromal oedema**	40	100*	50	100*	2	6.7
**Lymphocytic infiltration of the stroma**	39	97.5*	32	64.0*.**	0	0
**Alteration of epitheliocytes and glandular cells**	14	35.0*	12	24.0*.**	2	6.7
**Vascular sclerosis**	11	27.5*	13	26.0*	0	0
**Stromal necrosis**	2	5.0*	2	6.0*	0	0
**Thrombovasculitis**	4	10.0*	5	10.0*	0	0
**Moderate fibrosis of the fibrous structures of the stroma**	34	85.0*	35	70.0*.**	2	6.7
**Severe (fibroblastic transformation of a stroma)**	15	37.5*	11	22.0*.**	0	0
**Stromal calcifications**	24	60.0*	22	44.0*.**	0	0
**Decidual reaction**	0	0*	0	0*	23	76.7
**Glandular epithelium without signs of functional activity**	0	0*	0	0*	23	76.7
**Focal endometrial hyperplasia**	9	22.5*	4	8.0*.**	0	0
**Endometrial hyperplasia without atypia**	31	77.5*	46	92.0*	0	0

* – statistically significant difference compared to the control group, p<0.05; ** – statistically significant difference compared to Group1, p<0.05.

It should be noted that there was a significant percentage of such markers as edema of the endometrial stroma in all examined women from both groups. In 71 samples (78.9%), the lymphocytic infiltration of the stroma was diagnosed. At the same time, inflammatory infiltration in patients from Group 1 was noted significantly more often – by 1.5 times compared to women in Group 2 (p<0.05). The moderate fibrosis of the fibrous structures of the stroma was verified in 69 women (76.7%), fibroelastic transformation of the stroma of the endometrium was diagnosed in every third patient (26–28.9%). Normal characteristics of the endometrium were found in 30 patients from the control group: 28 of them (93.3%) had proliferative endometrium, 2 cases (6.7%) had secretory endometrium, and proliferative endometrium with changes (anovulatory endometrium) was revealed in 7 women (23.3%).

Compression sonoelastography allowed us to assess the course of the disease within the groups of patients more clearly. According to compression sonoelastography we identified 82 patients (91.1%) who had class II and III elastogram, characteristic of benign endometrial processes.

**Figure 2.**
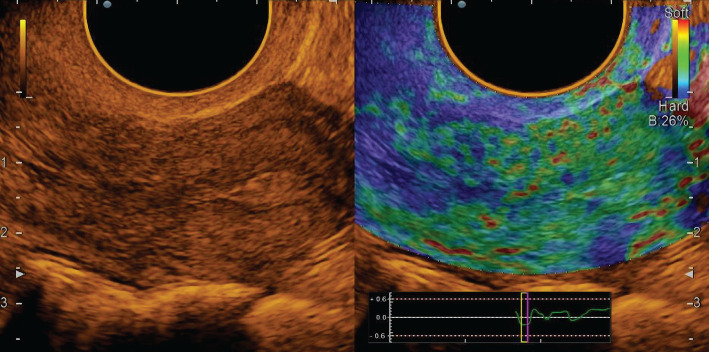
Endometrial polyp: polychromatic mode and compression sonoelastography mode (class II).

In all cases, endometrial polyps were mapped mainly in elastic green (class II elastogram), where the uterine cavity was outlined by a highly elastic red line (Figure 2).

Among the 25 cases of diagnosed polyps in most patients (7–28.0%), the polyp had a glandular-fibrous structure, and in combination with ultrasound angiography in the grayscale mode, it was visualized as small anechoic inclusions with vascular pedicle and blood supply. At the same time, in sonoelastography mode, it was plotted on the map as a mosaic. In 8 cases (32.0%), glandular polyp of the endometrium was detected, which for 6 patients (24.0%) in B-mode was defined as a weakly echogenic formation of a round shape with single vessels in ultrasound angiography. In 4 patients (16.0%), local thickening of the endometrium with deformation of the midline and the absence of pronounced vessels was visualized. In sonoelastography mode, the glandular polyp was mapped elastically with separate highly elastic inclusions and a contoured red line, which allowed to clarify the location of the polyp. The fibrous polyp was diagnosed in 6 patients (24.0%), and it was visualized as a rounded echogenic formation with a vascular pedicle in the grayscale mode. In contrast, in the sonoelastography mode, the mapping with individual dense areas of blue colour and the contour of the uterine cavity with a highly elastic red line was recorded.

**Figure 3.**
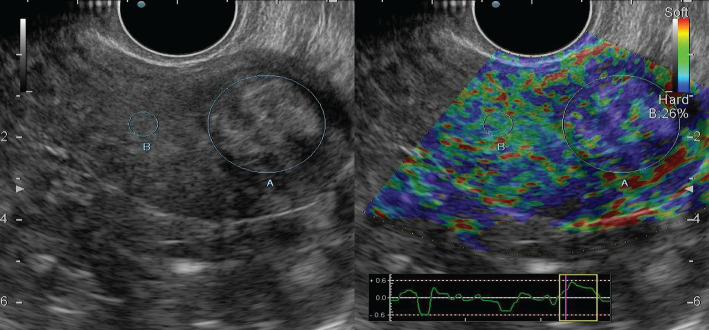
Endometrial hyperplasia: grayscale mode and compression sonoelastography mode (class II).

According to Figure 3, endometrial hyperplasia in the mode of compression sonoelastography was mapped elastically with shades of green colour, with linear highly elastic inclusions of red colour (class II elastogram).

**Figure 4.**
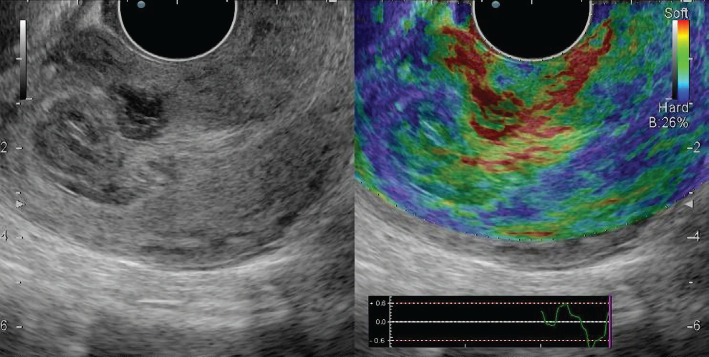
Submucosal node: grayscale mode and compression sonoelastography mode (class II).

In the case of a combination of HE and polyposis on the background of elastic mapping of the endometrium, mosaic areas were visualized; when combined with myomatous nodes, denser formations of blue shades were observed, which corresponded to the submucosal location of the node (Figure 4).

When performing elastography, in the projection of the myomatous nodule, staining in red shades was more often observed – in 5 (41.7%) cases, yellow – 2 (16.7%) and light blue – in 1 (8.3%) case; in 2 (16.7%) cases there was inhomogeneous red staining, and in 2 (16.7%) cases staining was inhomogeneous in red-yellow-light blue shades.

**Figure 5.**
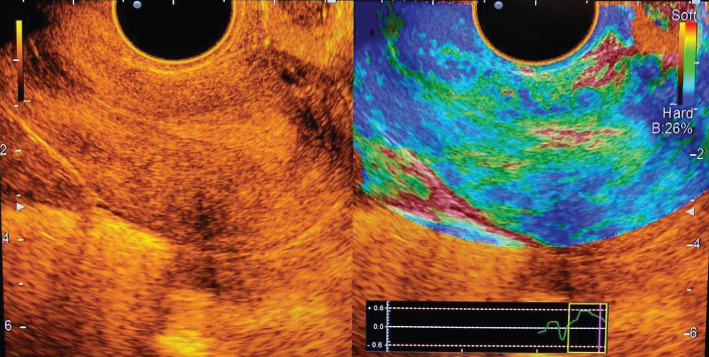
Normal endometrium: polychromatic mode and compression sonoelastography mode.

Unchanged myometrium in all patients from the control group was represented by blue shades, while staining was homogeneous in 27 (90.0%) patients (Figure 5).

**Figure 6.**
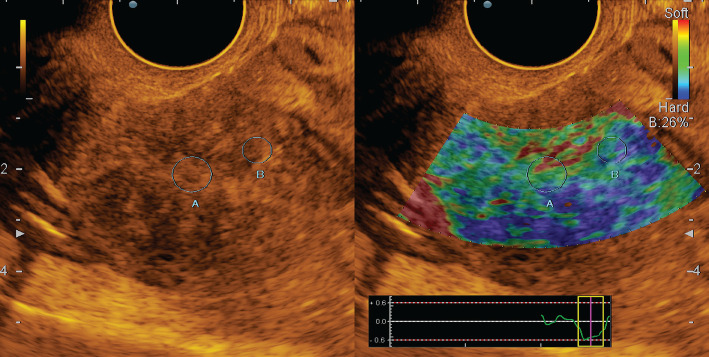
Chronic endometritis: polychromatic mode and compression sonoelastography mode (class II).

In patients with uterine factor infertility, compression sonoelastography allows not only to detect but also confirm the presence of tumors, assess their nature (sonographic signs of chronic endometritis) (Figure 6) and localization.

Additional information obtained in the case of the optimized diagnostic complex using compression sonoelastography allowed us to reduce the number of false-positive results in 86 cases (95.6%) and correctly interpret the nature of pathological changes in the endometrium and increase the sensitivity of ultrasound techniques.

## DISCUSSION

Endometrial hyperplastic processes are most common in the structure of intrauterine pathology and range from 30 to 55%. These have a negative impact on the effectiveness of ART programs and increase the implantation failure of the endometrium in 47% of cases [1, 2, 8]. Despite the improvement of diagnostic and treatment methods, in recent years, there has been an increase in the incidence of HE and polyposis, with up to 40% of women undergoing surgical treatment, often with loss of reproductive function [4, 5]. Therefore, the problem of treatment and prevention of HPE in reproductive age remains relevant, given the significant prevalence, propensity to relapse, lack of pathognomonic specific symptoms, the complexity of diagnosis, and choice of therapeutic programs [6, 10, 29, 30].

The problem of ovarian tumors in all age groups of women also remains urgent. According to various authors, their frequency increases from 11% to 19–25% in the structure of gynecological pathology [13, 17–19]. Most ovarian tumors (68–85%) are benign, including epithelial tumors that are considered the most common (48–76%). The combination of endometrial, myometrial, and ovarian pathology remains controversial in the literature, where some scientific reports noted changes in endometriosis, adenomyosis, and submucosal fibroids in 61% of patients with retention cysts and ovarian tumors of all age groups [8, 17–20]. In the case of true benign ovarian tumors, intrauterine pathology was found in 55%, in the case of retention cysts – in 69.4% with a predominance in the pre-and postmenopausal period [13, 17–20]. Many authors note performing intrauterine diagnostic manipulations (hysteroscopy, separate diagnostic scraping) in every fifth patient with ovarian pathology, and the results of histological examination confirmed endometrial hyperplasia in 88.9% of cases and glandular polyp – in 56.5% of patients [20]. Chronic inflammatory diseases of female genitals, menstrual disorder, surgical treatment of pelvic organs, hyperplastic processes of the myometrium are especially important in the occurrence of ovarian tumors [18–20].

Today, the increasing frequency of comorbidity is being actively discussed, which, without a doubt, has an additional negative impact on the course and progression of endometrial pathology. Endometrial hyperplastic processes are registered in almost every third patient in groups with uterine fibroids (38.1%), adenomyosis (39.2%), in every second patient with a combination of three nosologies (43.5%). Endometrial polyps were found more often: practically in two-thirds (61.3%) with certain nosology and in every second woman (56.4%) with their combination [4, 5, 7, 12, 19].

The contradictory nature of most aspects of the etiology and pathogenesis of HPE is caused by the combined influence of endocrine, immunological, hormonal, and genetic factors. Recent studies presented systemic and local hyperestrogenism, the duration of hormonal exposure, and the activity of the receptor apparatus of uterine tissues. The theory of chronic inflammatory processes characterized by a distortion of the effect of estrogen on the endometrium and inhibition of the functional activity of neutrophils causing immunological imbalance is becoming more relevant. However, the influence of infectious factors on the development of HPE remains poorly understood [2, 4, 5, 7, 12]. There are studies on the possibility of developing hyperplastic processes against the lack of hormonal disorders, which indicates other mechanisms of HE development [12]. Most studies consider chronic inflammation of the endometrium as a favorable background for developing dysregenerative hyperplastic and neoplastic diseases of the endometrium, which reveal changes in angioarchitectonics by endometrial inflammation tissue, processes of sclerosis and hyalinosis, violation of the normal cyclic transformation of the functional layer [9, 11, 21]. According to Shurshalina *et al.*, along with the change in the receptivity of the endometrium, the processes of proliferation and apoptosis are disturbed. There is an imbalance in the synthesis of proinflammatory cytokines and growth factors, accompanied by dysfunction of steroidogenesis [9].

In contrast to HE, estrogen dependence of endometrial polyposis is also questionable, and the inflammatory theory comes to the fore, which has put forward a scientific position on the existence of two different pathogenetic types of endometrial polyposis – hormone-dependent and inflammatory ones [12, 13, 17–19], in which both bring changes in the immune system, inflammatory factors and hormonal disorders [3, 10, 14–16]. In the literature, the influence of persistent chronic inflammatory process of both bacterial and viral etiology on the development of female proliferative diseases is discussed, which activates angiogenesis, alteration of tissue by mast cells, pathological proliferation, and initiates further progression of endometrial pathology [11, 21, 24, 26]. The etiological theory of polyp formation is ambiguous; issues of their prevention remain controversial when, according to scientific studies, the growth of polyps may be caused by the ovarian pathology (polycystic ovary syndrome, chronic inflammatory process of the uterine appendages, stromal tekomatosis), which can stimulate and support proliferative processes in the endometrium [11, 21, 24, 25].

Given innovative technologies, compression sonoelastography has been widely used recently, based on the principle of assessing the deformation of body tissues due to ultrasonic waves and low mechanical compression sensor, which provides a real-time assessment of tissue elasticity.

According to the evaluation results of a complex ultrasound examination with the inclusion of compression sonoelastography, patients with concomitant gynecological pathology showed a high sensitivity of this diagnostic complex and the ability to reduce the number of false-positive results. Publications using this technology in the algorithm of uterine examination are inconsiderable in number in the available literature nowadays. Among them, there are studies evaluating the Young modulus of the uterus in normal and pathological conditions, publications on the assessment of endometrial and myometrial elasticity in healthy women and the diagnosis of uterine tuberculosis, data on the analysis of morphological changes in the structure of the myometrium in adenomyosis [24, 25, 28]. This study demonstrates the feasibility of this diagnostic method for patients with uterine factor infertility.

## CONCLUSIONS

In the case of a combination of endometrial hyperplasia and ovarian cystic changes, a high percentage of comorbidity of gynecological pathology is verified. The frequency of hyperplastic endometrial processes in their combination (endometrial hyperplasia, uterine leiomyoma, adenomyosis, and polyposis) equals 37.8%. The concomitant chronic inflammatory process, menstrual disorder, and the comorbidity of gynecological pathology significantly complicate the ultrasound diagnosis of endometrial pathology.

Scientists believe that the development of hyperplastic endometrial processes in this category of patients is caused by a combination of two different pathogenetic mechanisms – hormone-dependent and inflammatory ones. Changes in the immune system, inflammatory factors, and hormonal status disorders that contribute to chronic endometritis with reactive endometrial hyperplasia are involved.

The conclusion of ultrasonic compression sonoelastography is an additional method of examination; it allows to detect pathological changes in the endometrium and uterine cavity at an earlier stage, obtain more complete information and differentiate hyperplastic processes, assess the severity of the lesion, clarify the state of the myometrium in leiomyoma and adenomyosis with a clear localization of the process, which undoubtedly expands its diagnostic capabilities.

## ACKNOWLEDGMENTS

### Conflict of interest

The authors declare that there is no conflict of interest.

### Ethical approval

This study was approved by the Ethics Committee of the HSEEU Ivano-Frankivsk National Medical University, Ivano-Frankivsk, Ukraine (approval ID: 123/21 - 21.09.2021).

### Consent to participate

Written informed consent was obtained from the patients.

### Data availability

Further data is available from the corresponding author on reasonable request.

### Authorship

OIK, MOM contributed to data analysis and wrote the original draft. MOM, HNI contributed to the methodology. HNI, HHM contributed to data collection. OOM, HNI contributed to editing the manuscript. HHM contributed to conceptualizing the study
